# Thermophoretic collection of virus-laden (SARS-CoV-2) aerosols

**DOI:** 10.1063/5.0039247

**Published:** 2021-03-12

**Authors:** Xiangzhi Zhang, Jing Wang, Chengbo Wang, Zheng Lian, Yong Shi, Yong Ren, Yuying Yan

**Affiliations:** 1 Research Group for Fluids and Thermal Engineering, University of Nottingham Ningbo China, Ningbo 315100, China; 2 Department of Mechanical, Materials and Manufacturing Engineering, University of Nottingham Ningbo China, Ningbo 315100, China; 3 Department of Electrical and Electronic Engineering, University of Nottingham Ningbo China, Ningbo 315100, China; 4 Key Laboratory of More Electric Aircraft Technology of Zhejiang Province, University of Nottingham Ningbo China, Ningbo 315100, China; 5 HiFiBio (Hangzhou) Co., Ltd., Hangzhou 311215, China; 6 Key Laboratory of Carbonaceous Wastes Processing and Process Intensification Research of Zhejiang Province, University of Nottingham Ningbo China, Ningbo 315100, China; 7 Research Group for Fluids and Thermal Engineering, University of Nottingham, Nottingham NG7 2RD, United Kingdom

## Abstract

Detecting the existence of SARS-CoV-2 in the indoor atmosphere is a practical solution to track the prevalence and prevent the spread of the virus. In this work, a thermophoretic approach is presented to collect the novel coronavirus-laden aerosols from the air and accumulate to high concentrations adequate for the sensitivity of viral RNA detection. Among the factors, the density and particle size have negligible effects on particle trajectory, while the vertical coordinates of particles increase with the rise in heating source temperature. When the heating temperature is higher than 355K, all of the particles exit the channel from one outlet; thus, the collecting and accumulating of virus-laden aerosols can be realized. This study provides a potential approach to accelerate the detection of SARS-CoV-2 and avoid a false negative in the following RNA test.

## I. INTRODUCTION

The outbreak of Coronavirus disease 2019 (COVID-19) pandemic has caused a dramatic impact on healthcare services and economies in the affected countries.^1,2^ The underlying pathogen has been confirmed to be a novel coronavirus, which was named as severe acute respiratory syndrome coronavirus (SARS-CoV-2) by the International Committee on Taxonomy of Viruses.^3^ As reported, the aerosolized virus particles carried by humans are caused by coughing/sneezing, even normal breathing or speech of an infected person.^4^ The disease is asseverated to be transmitted by multiple pathways, including direct (deposited on persons) or indirect (deposited on objects) contact and airborne transmission.^5^ Having analyzed the prevalence trends in China, Italy, and the United States from January 23 to May 9, 2020, Zhang *et al.*^6^ illustrated that airborne transmission is the dominant route to spread the disease. Respiratory particles are the media of airborne transmission, which are commonly distinguished to be droplets or aerosols based on their aerodynamic diameter.^7^ According to the CDC of the United States, the particles of more than 5μm are categorized as droplets and those less than 5μm as aerosols or droplet nuclei.^8^ A study conducted by Papinei *et al.*^9^ showed that 80%– 90% of particles generated by human expiratory activities were aerosols, most of which were generated during coughing and least of which from nasal breathing. Up to now, the transmission mechanisms of aerosols within confined spaces are still complex and remain to be studied, especially for the indoor environment.^10^ Highly dispersed in aerosols, the virus can stay viable and infectious for several hours.^11^ Under the condition of long exposure to high concentrations of aerosols, inhaled aerosols containing virus can deposit directly along the human respiratory tract, which causes infection in the alveolar tissues of the lower respiratory tract.^12^

An aerosol is defined as a suspension system of solid or liquid particles in the air or another gas.^7^ Airborne transmission can be achieved via aerosols carrying viruses, e.g., the influenza A H1N1,^13^ severe acute respiratory syndrome (SARS),^14^ and middle east respiratory syndrome (MERS).^15^ The indoor aerosols are most broadly defined as ultrafine ( <0.1μm), fine ( 0.1∼2.5μm), or coarse ( >2.5μm).^16^ Driven by Brownian motion, some of the virus-laden aerosols diffuse toward lateral directions which results in long-distance nosocomial transmission in the confined space. The transmitting payloads and environmental tolerance of SARS-CoV-2 virus of the indoor environment depend on factors including the specific phenotype available, the composition of the aerosols, and the physical characteristics of the surrounding environment.^13^ Liu *et al.*^17^ investigated the generation of airborne SARS-CoV-2 and the aerosol deposition at 30 sites in two designated hospitals in Wuhan and found the concentrations of airborne SARS-CoV-2 (ranging from 16 to 42 copies per $m^{3}$ ) in the protective-apparel removal rooms were among the upper range.

Keeping social distancing is expected to be effective to prevent infection via bioaerosol contact.^18^ Besides, precautions against airborne transmission in indoor scenarios should be taken including increasing ventilation rate, using natural ventilation, avoid air recirculation, avoiding staying in another person’s direct airflow, and minimizing the number of people sharing the same environment.^19^ Except for precautions, reliable diagnosis is important for epidemic prevention and control of the virus. At present, several molecular assays that detected the COVID-19 have been developed and recognized by the WHO.^20^ Among them, the reverse transcription polymerase chain reaction (RT-PCR) is commonly employed to detect the viruses and the sensitivity to detect the RNA-dependent RNA polymerase (RdRp) sequence is about 3.7 RNA copies.^21^ However, the RT-PCR assays for virus detection has reported cases of false-negative results since its amplification of spurious nucleic acid contamination, and it is difficult to directly detect the viruses travelling in the air since sampling and detecting of the presence of SARS-CoV-19 are time-consuming.^5^ The objective of this study is to present a novel microfluidic method to collect virus-laden aerosols from the indoor air, which enables improved sensitivity compared with existing viral detecting methods.

## II. PROBLEM DESCRIPTION AND CONFIGURATION

Due to a study conducted in Singapore,^22^ high viral RNA-contained aerosols ( $1.84\times 10^{3}-3.38\times 10^{3}$ RNA copies per $m^{3}$ air) were detected in airborne infection isolation rooms, and surface contamination was also detected in rooms with virus-contained aerosols. The minimum size of SARS-CoV-2 is about 60 nm, but the combined size of virus-laden aerosol can be larger than 100 nm when attached to a larger carrier aerosol.^2^ Aerosols suspended in air collide and merge to become larger, while the shear force breaks them up. Due to the effects of coalescence and breakup, the equilibrium size is approximately 80 nm in heavy traffic area, while it can be below 100 nm in water vapor under high humidity.

**FIG. 1.**
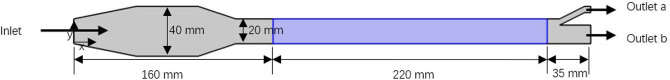
Structure of the device. The length of the channel is 415 mm, including an inlet region of 160 mm, a heated region of 220 mm, and an outlet region of 35 mm.

In this study, a 2D model of the device is considered to investigate the performance of thermophoretic separation and collection of virus-laden aerosols. The main component of the device is a channel, and the bottom wall of which is connected with a heating source. Driven by the thermophoretic force, the particles will move upward and exit the channel through the upper outlet. The length of the channel is 415 mm, including an inlet region of 160 mm, a thermophoresis region of 220 mm, and an outlet region of 35 mm. The geometric structure of the device is shown in Fig. 1.

In the evaluation of RNA stability of SARS-CoV-2 under thermal treatment, the virus was inactivated after being heated at $60{\;}^{{}^{\circ}}$C for more than 15 min,^23^ which allows the thermophoretic sorting of virus-laden aerosols during a short time interval.

## III. MATHEMATICAL MODEL

### A. Thermophoretic force

Thermophoresis is an important transport mechanism of small particles in a non-isothermal carrier fluid driven by the temperature gradient. Particles suspended in a non-isothermal mixture subject to a force pushing them in the direction of the temperature drop. The driving mechanism behind this force is the collision of gas molecules on the particles’ surface. Collisions are more likely to occur on the hotter side of the particle where the average molecular velocity of the gas is greater. This results in a net force toward colder regions of the gas. Particle with different physical properties exhibit different responses to the force, which realizes sorting and separation. In gas media, the thermophoretic force acting on a suspended particle depends on the flow regime characterized by the Knudsen number Kn=λ/L, where λ is the mean molecular free path and L is the characteristic length of the particle. For small particle or large molecular mean free path when Kn≫1, the effect of the particle motion on the distribution of the fluid molecular velocities can be virtually neglected. Solving the Boltzmann equations in continuum ( Kn≪1) and transition ( Kn≈1) regimes where the velocity distribution of molecules is greatly affected by the movement of particles presents considerable complexities, since rarefied gas dynamics remains to be resolved by kinetic theory. Epstein^24^ derived an equation for the thermophoretic force exerted on spherical particles in gases based on a continuum analysis,

$$F_{tp}=-\frac{4.5\pi d_{\;p}\mu^{2}}{\rho }\frac{1}{2+\frac{k_{p}}{k}}\frac{\nabla T}{T},$$ (1)

where $d_{\;p}$ is the diameter of the particle, μ is the coefficient of shear viscosity, ρ is the mass density, $\frac{k_{p}}{k}$ is the thermal conductivity ratio of particle and fluid, and T is the absolute temperature. Since boundary conditions appropriate for the slip-flow regime have not been used and the continuum energy equation has been solved regardless of the convective terms, serious disagreement between Eq. (1) and experimental results.^25^ Brock^26^ conducted a hydrodynamic analysis for small Kn≪1 in a near continuum regime and developed a general equation with introducing matching coefficients associated with the temperature jump and velocity slip. Talbot *et al.*^27^ established an equation to describe the thermophoretic force for the entire range of Kn,

$$F_{tp}=-\frac{6\pi \mu^{2}d_{\;p}C_{s}\left(\frac{k}{k_{\;p}}+C_{t}\frac{2\lambda }{d_{\;p}}\right)\frac{\nabla T}{T}}{\rho \left(1+6C_{m}\frac{\lambda }{d_{\;p}}\right)\left(1+2\frac{k}{k_{\;p}}+4C_{t}\frac{\lambda }{d_{\;p}}\right)},$$ (2)

where the matching parameters $C_{s}=1.17$, $C_{m}=1.14$, and $C_{t}=2.18$. According to the Stokes expression, the thermophoretic velocity is obtained as

$$u_{tp}=-\frac{2\eta C_{s}\left(\frac{k}{k_{\;p}}+C_{t}\frac{2\lambda }{d_{\;p}}\right)\frac{\nabla T}{T}}{\rho \left(1+6C_{m}\frac{\lambda }{d_{\;p}}\right)\left(1+2\frac{k}{k_{\;p}}+4C_{t}\frac{\lambda }{d_{\;p}}\right)},$$ (3)

where η is the dynamic viscosity of the gas.

### B. Governing equations and boundary conditions

This model is composed of flow field, temperature field, and particle tracing. The flow field is characterized by Navier–Stokes equations,

$$\left\{ \begin{array}{c} \nabla \cdot (\rho u)=0\;\\ \rho (u\cdot \nabla )u=\nabla \cdot [-pI+\mu \left(\nabla u+{\left(\nabla u\right)}^{⊤}\right)-\frac{2}{3}\mu (\nabla \cdot u)I]+F \end{array}\right.,$$ (4)

where ρ is the density, u is the velocity, p is the pressure, I is the identity matrix, μ is the viscosity, and F is the force term.

Heat transfer in fluids is characterized by

$$\rho c_{\;p}\frac{\partial T}{\partial t}+\rho c_{\;p}u\cdot \nabla T+\nabla \cdot (-k\nabla T)=Q,$$ (5)

where $c_{\;p}$ is the heat capacity, T is the temperature, and Q is the heat flux term.

The trajectory of the particle in the fluid flow is characterized by

$$\frac{d\left(m_{\;p}u\right)}{dt}=F_{p},$$ (6)

where $F_{p}$ is the force of particles. Particles in aerosols are often subjected to Brownian motion, gravity, electrostatic forces, thermal gradients, electromagnetic radiation, turbulent diffusion, and inertial forces.^28^ Buongiorno *et al.*^29^ investigated the relative effects of inertia, Brownian diffusion, thermophoresis, diffusiophoresis, the Magnus effect, fluid drainage, and gravity that may cause a relative motion of particles in the main fluid. For particles at nanoscale, only thermophoresis and Brownian diffusion can cause slip. For micro-sized particles, gravity (weight and buoyancy) should be considered, while Brownian diffusion is less important. In continuum mechanics, the Froude number is a dimensionless number defined as the ratio of the flow inertia to the external field, expressed as

$$Fr=\frac{u}{\sqrt{gL}},$$ (7)

where u is the magnitude of local flow velocity, g is the magnitude of the gravity field, and L is a characteristic length. In this study, the Froude number Fr≪1 and the flow in the channel is a subcritical flow;^30^ thus, the source term in Eq. (6) can be expressed as

$$F_{p}=F_{b}+F_{g}+F_{d}+F_{tp},$$ (8)

where $F_{b}$, $F_{g}$, $F_{d}$, and $F_{tp}$ represent the Brownian force, gravity force, drag force, and thermophretic force, respectively.

Brownian motion is the random, uncontrolled movement of particles in a fluid as they constantly collide with other molecules, which leads to spreading of particles from regions of high particle density to low density.^31^ The total force on the particles that undergo Brownian motion is expressed by a Brownian force term $F_{b}$,

$$F_{b}=\zeta \sqrt{\frac{12\pi k_{B}\mu Tr_{p}}{\Delta t}},$$ (9)

where ζ is a normally distributed random number with a mean of zero, $k_{B}=1.380\;649\times 10^{-23}\;J/K$ is the Boltzmann constant, μ is the fluid dynamic viscosity, T is the absolute fluid temperature, $r_{p}$ is the particle radius, and Δt is the time step taken by the solver. Gravity force is expressed by

$$F_{g}=m_{\;p}g\frac{\rho_{\;p}-\rho }{\rho_{\;p}},$$ (10)

where $m_{p}$ is the mass of particle, g is the gravity vector, and $\rho_{p}$ is the density of the particle. $F_{d}$ represents the drag force expressed as

$$F_{d}=\frac{18\mu }{\rho_{p}d_{\;p}^{2}}m_{\;p}u_{r},$$ (11)

where $\rho_{p}$, $d_{\;p}$ represents the density or diameter of the particle, respectively; $u_{r}=u-u_{\;p}$ is the relative velocity. And the thermophoretic force exerted on aerosol particles is expressed as Eq. (2).

Air and aerosols are injected into the domain, carried by an air flow of 2000 $Q_{SCCM}$. The standard molar volume $V_{m}=0.022\;413\;6\;m^{3}/\mathrm{mol}$ and the mean molar mass $M_{n}=0.002\;\mathrm{kg}/\mathrm{mol}$. The diameter of the inlet is 20 mm, with temperature 293.15 K. The boundary condition of the outlet has a relative pressure p=0. After entering the thermophoresis section, the particles will be heated by a susceptor (with temperature $T_{susc}$) and then migrate to the upper wall due to the impact of thermophoresis. The heat convection with the environment ( $T_{e}=293.15\;K$) is through the other walls (thickness d=5mm, the heat transfer coefficient $h=10\;W/m^{2}K$).

## IV. RESULTS AND DISCUSSION

### A. Numerical validation

The numerical simulation is conducted on a desktop PC with Intel(R) Core(TM) i7-6700 CPU and 32 GB physical RAM. Table I shows the number of elements, minimum element quality, and the number of degree of freedom (DOF) with varying grid densities. According to this table, the minimum element quality is greater than 0.1 when the number of domain elements is greater than 1286. Therefore, numerical simulation is conducted on the mesh with 884 boundary elements and 15 718 domain elements.

**TABLE I.** Elements numbers, minimum element quality, and degree of freedom (DOF) with varying grid densities.

Boundary elements	Domain elements	Minimum element quality	Degree of freedom(DOF)
211	1 286	0.1849	3 802
305	2 134	0.1633	6 083
382	3 311	0.2029	8 930
559	6 187	0.2021	15 808
707	9 415	0.1877	23 210
884	15 718	0.2052	36 927
1820	41 019	0.2144	93 571
3484	104 354	0.2117	230 995
3516	162 422	0.2074	347 199

**FIG. 2.**
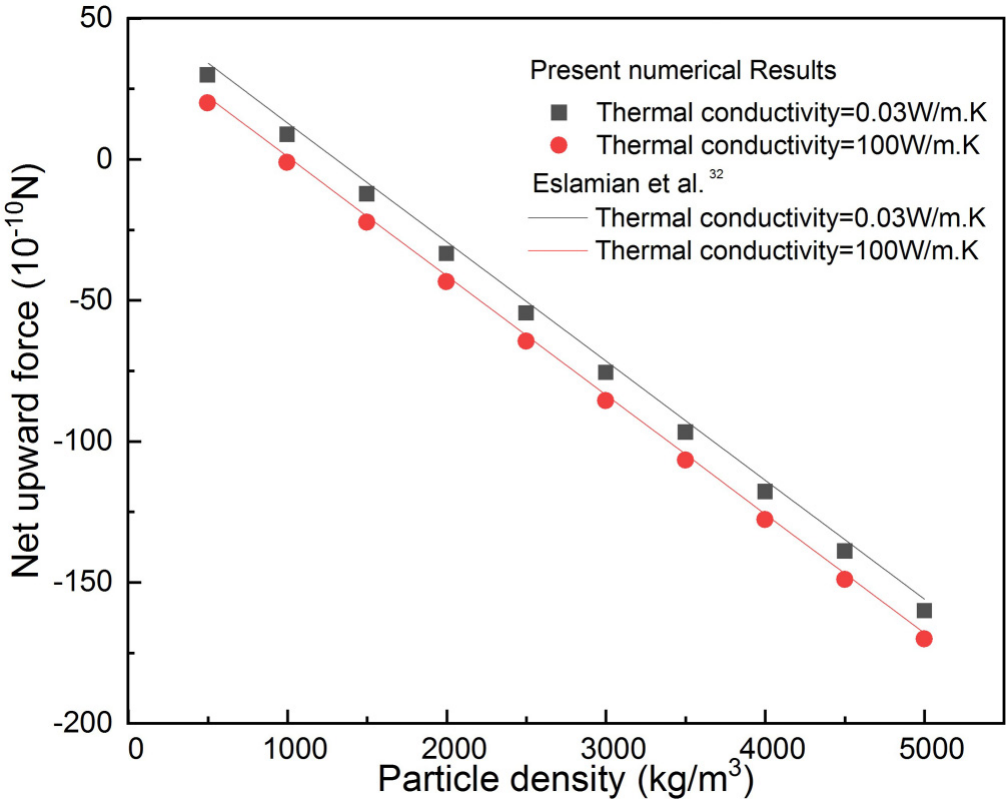
Comparison of net upward force variations along particle density.

The validation of the model presented in this study is investigated by comparing with the results of Eslamian *et al.*^32^ for precise particle thermophoretic separation and manipulation in microchannels. In their study, two-dimensional microchannel designs with a width of 500μm and length of 8 mm with one inlet and two outputs are considered. Figure 2 shows that the agreement of results is generally well.

### B. Fields and thermophoresis performance

**FIG. 3.**
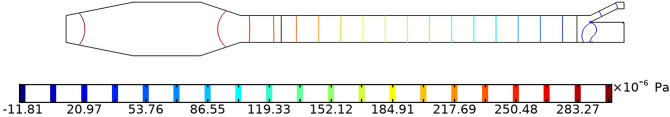
Pressure distribution in the channel with the air flow of $2000Q_{SCCM}/m$ at the inlet.

**FIG. 4.**
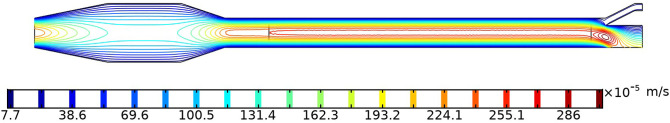
Distribution of velocity magnitude with the air flow of $2000\;Q_{SCCM}/m$ at the inlet.

**FIG. 5.**
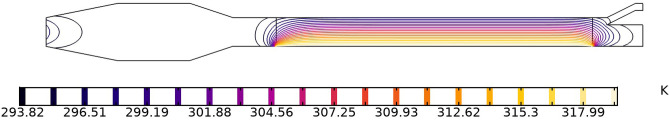
Distribution of temperature when $T_{susc}=320$ K.

In this study, the numerical simulations are conducted to investigate the particle trajectories of aerosols in a laminar flow of carrier fluid in a channel shown in Fig. 1. The flow of air in the channel with aerosol particles injected is shown in Figs. 3 and 4. According to Fig. 3, the global pressure drop is $3.28\times 10^{-4}$ Pa. The maximum Reynolds number is approximately 0.05, and the flow regime in this channel is the creeping flow. As shown in Fig. 4, the average radial velocity $u_{a}=0.0014\;m/s$. The flowrate at the upper outlet: $v_{a}=4.9412\times 10^{-7}\;m^{2}/s$, while the flow rate at the lower outlet: $v_{b}=1.6076\times 10^{-5}\;m^{2}/s$. The ratio of the outlet flowrate $v_{a}/v_{b}=0.028\;87$, and the particle concentration is raised at approximately 35 times. The temperature distribution when the bottom wall is connected with a heating source of 320K is shown in Fig. 5.

**FIG. 6.**
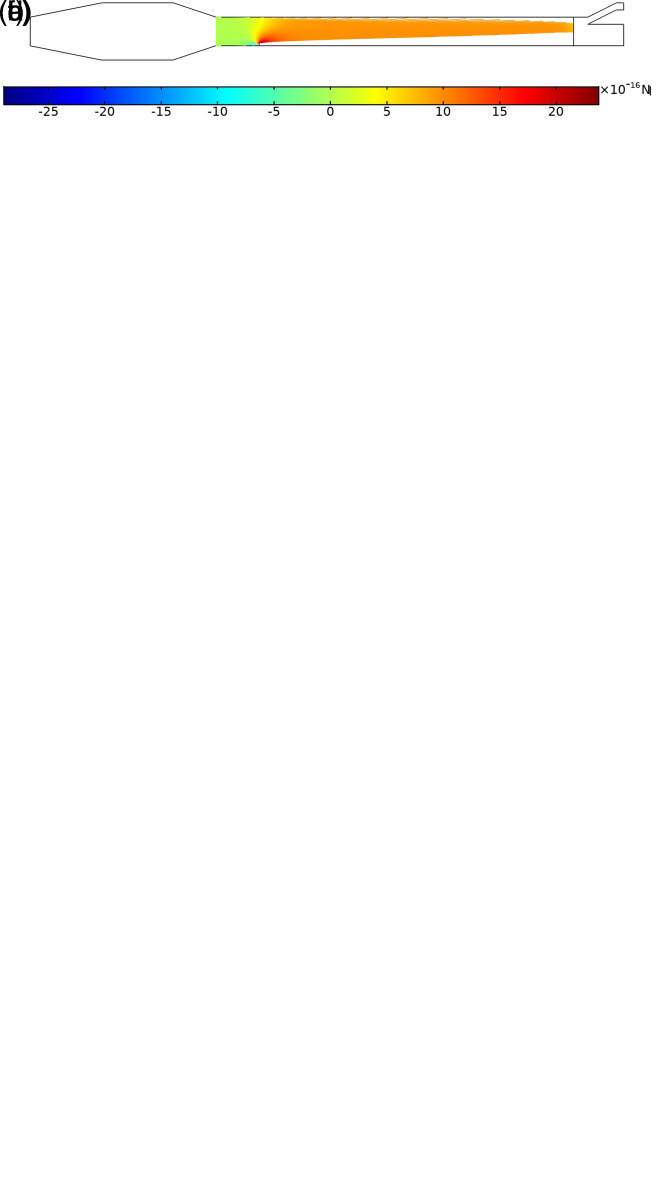
Real-time positions of aerosol particles when $T_{susc}=320\;K$, the color legend represents the vertical component of thermophoretic force, (a) t=10 s, (b) t=30 s, (c) t=50 s, (d) t=70 s, (e) t=90 s, and (f) t=110 s.

**FIG. 7.**
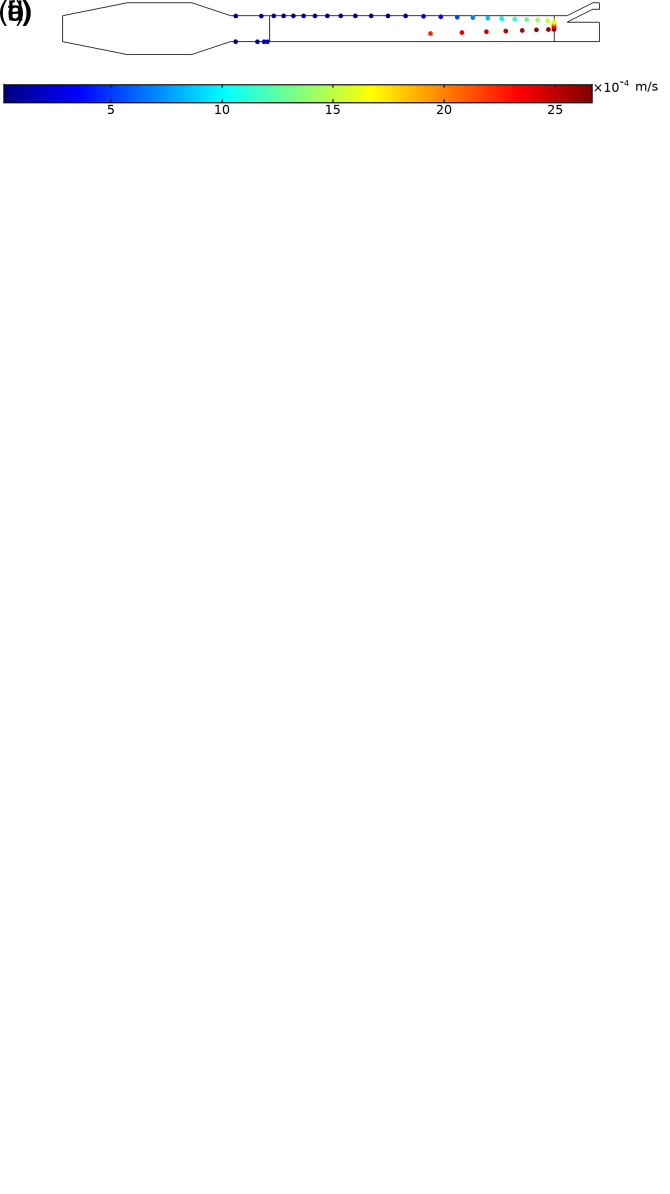
Real-time positions of aerosol particles when $T_{susc}=320\;K$, the color legend represents the vertical velocity, (a) t=10 s, (b) t=30s, (c) t=50s, (d) t=70 s, (e) t=90 s, and (f) t=110 s.

The real-time trajectories of aerosol particles are shown in Figs. 6 and 7.

**FIG. 8.**
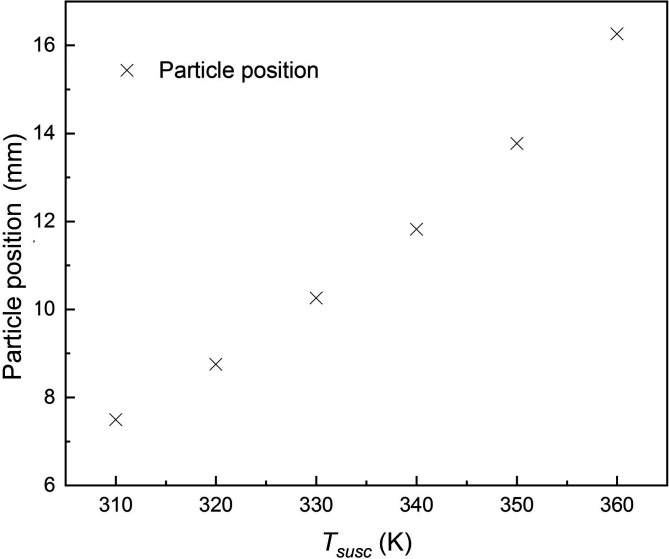
The minimum vertical coordinates of particles at the outlet with heating source temperatures.

Due to the thermal gradient, aerosols are driven by thermophoretic force to move upward and then exit the channel through the upper outlet. On the horizontal direction, the particles are carried by the inertia force and move right with the carrier fluid. On the vertical direction, the particles are driven by buoyancy and thermophoretic forces to move upward, whereas the gravity acts downward. Gravity and buoyancy forces are proportional to the particle diameter cubed, while the thermophoretic force is linearly proportional to the particle diameter. Since the density of aerosol particles is close to the carrier fluid, the buoyancy can be almost balanced by gravity. Therefore, the trajectories of particles with varying sizes showed a tiny divergence on the vertical direction, while the density and particle size have negligible effect on particle trajectory. According to Eq. (6), thermophoretic force is also determined by the local temperature and its gradient. The minimum vertical coordinates (infima) of aerosols at the outlet with varying heating source temperatures are shown in Fig. 8.

According to Fig. 8, the vertical coordinates of aerosols increase with the rise in heating source temperature. When the temperature is higher than 355 K, the minimum vertical coordinate of particles is 15 mm, which means all of the particles exit the channel from the upper outlet; thus, the collecting and accumulating of virus-laden aerosols are achieved. The concentration of virus-laden aerosols can be raised to 25 times after thermophoretic sorting, thus enhancing the RNA concentration, which can be beneficial to reduce the possibility of false negative in the RNA test.

## V. CONCLUSION

In this study, thermophoresis is adopted as a novel technique to collect and accumulate virus-laden aerosols from indoor air. Numerical simulations were conducted by coupling the transfer equations of heat and mass transfer with particle tracing for the fluid flow. The availability of this device was verified and suitable operation conditions were determined based on the simulation and the parameters acquired from articles and experiments. It has been acquired that thermophoretic force is significant to drive the virus-bearing aerosols to move upward and accumulate at the cold side. When the heating temperature is higher than 355 K, all the particles exit the channel through the upper outlet, thus achieving the collecting of virus-laden aerosols. Considering the stability of viral RNA, a suitable range ( 360−380 K) of heating source temperature is acquired. Under these operation conditions, the aerosols can be collected from indoor air and accumulated to a higher density that is beyond the test requirements without being destroyed by heat. This technique provides a method to reduce the time interval of the RNA test of SARS-CoV-2 and avoid a false negative for RNA detection, which is helpful to suppress the spread of the pandemic of COVID-19.

## AUTHORS’ CONTRIBUTIONS

A.X.Z. and F.Y.R. proposed the research and designed the numerical work; A.X.Z. conducted the simulation; A.X.Z. and F.Y.R. wrote the manuscript; and B.J.W., C.C.W., D.Z.L., E.Y.S., and G.Y.Y. contributed to the discussion of the data and review of the manuscript.

## Data Availability

The data that support the findings of this study are available from the corresponding author upon reasonable request.
